# Personal

**Published:** 1896-02

**Authors:** 


### Personal.

DR. WILLIAM PERRY WATSON, of Jersey City, has resigned the secretaryship of the New Jersey state medical examining and licensing board. Dr. Watson also retires from the practice of medicine to accept the medical directorship of a large life insurance company. Dr. Watson will bring great experience to his new field, but also will be greatly missed in the state examining board. Dr. E. L. B. Godfrey, of Camden, has been elected secretary to fill the vacancy.

---

Dr. A. C. Abbott, of Philadelphia, first assistant in the laboratory of hygiene at the University of Pennsylvania, by invitation addressed the section for pathology of the Buffalo Academy of Medicine, January 21, 1896, on the Principles of preventive inoculation and serum therapeutics, with considerations concerning their practical application. This paper will be published in an early issue of the Journal.

---

Mr. George W. Rafter, of Rochester, a member of the state engineering corps, read a paper before the Microscopical club, January 13, 1896, entitled Lake Erie as a water supply for the towns on its borders.

---

Dr. J. Henry Dowd, of Buffalo, has removed his office from 166 Broadway to 206 Franklin street. His practice is limited to genito-urinary surgery, syphilis and skin diseases. Hours, 9–11 ; 7–8 ; Sunday, 2–4.

---

Dr. George L. Brown, of Buffalo, has removed his office from 121 Franklin street to the D. S. Morgan building, corner of Pearl and Eagle streets. Hours, 9–11 ; 1–4 ; Sunday, 10—1.

---

Dr. Chauncey Pelton Smith, of Buffalo, will read a paper before the Microscopical Club on the Etiology of cancer, February 10, 1896.

